# Editorial: The CRISPR/Cas System in Pathogen Resistance, Virulence, Diagnosis and Typing

**DOI:** 10.3389/fmicb.2022.832152

**Published:** 2022-03-03

**Authors:** Guangcai Duan, Biao Kan, Dongsheng Li, Hongbin Song

**Affiliations:** ^1^Department of Epidemiology, College of Public Health, Zhengzhou University, Zhengzhou, China; ^2^National Institute for Communicable Diseases Prevention and Control, Chinese Center for Disease Control and Prevention, Beijing, China; ^3^Department of Cell and Molecular Biology, QIMR Berghofer Medical Research Institute, Herston, QLD, Australia; ^4^Center for Disease Control and Prevention of PLA, Beijing, China

**Keywords:** CRISPR/Cas systems, antimicrobial resistance, virulence, diagnosis, genotyping

## Introduction

CRISPR-Cas systems constitute an adaptive immunity in prokaryotes, empowering the bacteria with defense against invasive mobile genetic elements (Barrangou et al., 2007; Sorek et al., 2008). In the past 10 years, owing to the unprecedented application of the engineered CRISPR-Cas9 nucleases worked as tools of genome-editing, comparative genomics, biochemical activities as well as biological functions of CRISPR-Cas systems and individual Cas proteins have become a subject of intensive study (Sternberg et al., 2016; Koonin et al., 2017; Makarova et al., 2020). Therefore, this Research Topic concentrates on the advancement of CRISPR-Cas systems in developing new generation diagnostic platforms, typing applications and therapeutic approaches against microbial infections.

We sincerely appreciate all contributors who have submitted their excellent papers to our Research Topic. The current Research Topic provides an effective communication platform, collecting both original research articles examining explorations of CRISPR-based typing, CRISPR diagnostics, CRISPR-Cas diversity in S. aureus and healthcare-related pathogens, and review papers concerning CRISPR-Cas antimicrobials and CRISPR-based pathogen detection. This collection of 7 articles can be divided into three sections: Characterization of CRISPR-Cas systems in pathogens and typing applications; CRISPR-powered diagnostics; CRISPR-Cas systems in the battle against antimicrobial resistance.

## Characterization of CRISPR-Cas Systems in Pathogens and Typing Applications

Staphylococcus aureus (S. aureus) is one of main pathogenic factors of nosocomial and community-acquired infections and represents a significant burden on the healthcare system (Kwiecinski and Horswill, 2020). Wang et al. analyzed 67 confirmed CRISPR loci and 15 companion Cas proteins in 52 strains of Staphylococci. In this bioinformatics article, they introduced the distribution and structure of CRISPR-Cas system in S. aureus according to a comprehensive analysis of the available genomic database. As a result of this analysis, they found that unlike the orphan CRISPRs away from Staphylococcal cassette chromosome mec (SCCmec), the complete CRISPR-Cas systems were in J1 region of SCCmec in *S. aureus*. The results of this analysis provide new insight into the diversity and characterization of the CRISPR-Cas system in *S. aureus*. Cutibacterium acnes (*C. acnes*), a commensal skin bacterium, is considered as the most prevalent cause of prosthetic joint infection, particularly of the shoulders (Boisrenoult, 2018). Cobian et al. performed a comprehensive comparative analysis of 255 *C. acnes* genomes available at NCBI database. Their results indicated the fundamental differences among *C. acnes* phylotypes, and they also reported diverse type I-E CRISPR-Cas systems and prophage sequences in select clades, which provide insights into strain divergence and typing applications. Healthcare acquired infections are an important determinant of outcome for patients in the hospital settings (Kollef et al., 2021). Mortensen et al. annotated and compared the CRISPR–Cas systems in healthcare-related bacteria, and found a highly broad spectrum of CRISPR–Cas systems in these pathogens. Among them, only 0.55% of *S. aureus* isolates possesses CRISPR–Cas systems, whereas *C. difficile* isolates they analyzed have CRISPR-Cas systems each possessing diverse CRISPRs. More specifically, their statistical results suggest that CRISPR-Cas containing isolates tend to carry more antimicrobial resistance (AMR) genes for several pathogens such as *A. baumannii, E. faecium, P. aeruginosa*, and *S. aureus*. These results provide an important resource for developing potential clinical applications of the CRISPR-Cas systems to combat antibiotic resistant pathogens. Salmonella is one of the primary causes of morbidity and mortality in foodbornebacterial gastroenteritis in the world (Petersen and Miller, 2019). Li et al. analyzed the CRISPR arrays of 75 Salmonella isolates obtained from poultry farms in China, and found 517 unique spacer sequences and 31 unique direct repeat sequences. Based on the features of CRISPR spacer sequences they exploited a new typing method of CRISPR locus three spacer sequences typing (CLTSST) to identify sources of Salmonella outbreaks especially correlated with epidemiological data.

Taken together, these findings expand the emerging evolutionary insights of pathogenic bacteria, and our novel understanding of CRISPR-Cas characterization in these pathogens, which will be beneficial for developing CRISPR-based typing methods.

## CRISPR-Powered Diagnostics

Quantitative polymerase chain reaction (qPCR) is commonly the gold-standard method for many nucleic-acid-based diagnostics (Kaminski et al., 2021). However, the costs of reagents used for qPCR are always high, and the method requires sophisticated laboratory device and experienced personnel (Kaminski et al., 2021). By contrast, the recently developed CRISPR-based diagnostics have displayed advantages of high sensitivity, specificity, rapidness, convenience, and low cost and have the potential to meet those unmet needs (Chertow, 2018; Kaminski et al., 2021). Zhang et al. reviewed past improvements of CRISPR diagnostics. The comparison of traditional qPCR text with the new CRISPR diagnostics emphasized the difference in sensitivity and cost. Compared to traditional molecular diagnostic techniques such as qPCR, CRISPR-based diagnostics employ the single-stranded nucleic acid trans-cleavage activities of either Cas12 or Cas13, which show advantages in both sensitivity and specificity and thus possess great potentiality both in diagnosis of emerging infectious diseases and beyond. Therefore, CRISPR-based diagnostics have been widely thought as the next-generation diagnostic methods. Because of the lack of standardized definition of Cas trans-cleavage enzymatic units, it brings serious difficulties to standardize the present CRISPR- powered diagnostics systems, which have undoubtedly curbed the development of the CRISPR technological industry. Lv et al. optimized the reaction systems for Cas12a, and then defined its trans-cleavage units (transU), which will be of great importance and interest to researchers in both molecular diagnostic industry and basic research.

## CRISPR-Cas Systems in the Battle Against Antimicrobial Resistance

The emergence of AMR becomes a global public health concern that threatens our capability to overcome infectious diseases (McEwen and Collignon, 2018). It is worth noting that CRISPR-Cas systems can be purposed to precisely target antibiotic resistance genes and viral genomes based on key Cas effectors (Gholizadeh et al., 2020). Duan et al. summarized the development of CRISPR-Cas antimicrobials to eliminate AMR microbes and plasmids. However, the use of CRISPR-Cas antimicrobials remains at an extremely initial stage and numerous barriers await to be overcome.

In summary, this collection of 7 manuscripts covers a variety of topics in the CRISPR/Cas System in pathogen resistance, virulence, diagnosis and typing, and proposes future directions for research to accelerate advancement in these considerable but often understudied CRISPR/Cas systems.

## Author Contributions

All authors listed have made a substantial, direct, and intellectual contribution to the work and approved it for publication.

## Funding

The work was supported by the National Science and Technology Specific Projects (2018ZX10301407).

## Conflict of Interest

The authors declare that the research was conducted in the absence of any commercial or financial relationships that could be construed as a potential conflict of interest.

## Publisher's Note

All claims expressed in this article are solely those of the authors and do not necessarily represent those of their affiliated organizations, or those of the publisher, the editors and the reviewers. Any product that may be evaluated in this article, or claim that may be made by its manufacturer, is not guaranteed or endorsed by the publisher.
